# Automatic planning of MR-guided transcranial focused ultrasound treatment for essential tremor

**DOI:** 10.3389/fnimg.2023.1272061

**Published:** 2023-10-26

**Authors:** Jan Klein, Annika Gerken, Niklas Agethen, Sven Rothlübbers, Neeraj Upadhyay, Veronika Purrer, Carsten Schmeel, Valeri Borger, Maya Kovalevsky, Itay Rachmilevitch, Yeruham Shapira, Ullrich Wüllner, Jürgen Jenne

**Affiliations:** ^1^Fraunhofer Institute for Digital Medicine MEVIS, Bremen, Germany; ^2^Department of Diagnostic and Interventional Radiology, University Hospital Bonn, Bonn, Germany; ^3^Clinic and Policlinic for Neurology, University Hospital Bonn, Bonn, Germany; ^4^Clinic for Neuroradiology, University Hospital Bonn, Bonn, Germany; ^5^Clinic and Policlinic for Neurosurgery, University Hospital Bonn, Bonn, Germany; ^6^INSIGHTEC Ltd., Tirat Carmel, Israel

**Keywords:** essential tremor, transcranial focused ultrasound, fiber tractography, segmentation, therapy planning

## Abstract

**Introduction:**

Transcranial focused ultrasound therapy (tcFUS) offers precise thermal ablation for treating Parkinson's disease and essential tremor. However, the manual fine-tuning of fiber tracking and segmentation required for accurate treatment planning is time-consuming and demands expert knowledge of complex neuroimaging tools. This raises the question of whether a fully automated pipeline is feasible or if manual intervention remains necessary.

**Methods:**

We investigate the dependence on fiber tractography algorithms, segmentation approaches, and degrees of automation, specifically for essential tremor therapy planning. For that purpose, we compare an automatic pipeline with a manual approach that requires the manual definition of the target point and is based on FMRIB software library (FSL) and other open-source tools.

**Results:**

Our findings demonstrate the high feasibility of automatic fiber tracking and the automated determination of standard treatment coordinates. Employing an automatic fiber tracking approach and deep learning (DL)–supported standard coordinate calculation, we achieve anatomically meaningful results comparable to a manually performed FSL-based pipeline. Individual cases may still exhibit variations, often stemming from differences in region of interest (ROI) segmentation. Notably, the DL-based approach outperforms registration-based methods in producing accurate segmentations. Precise ROI segmentation proves crucial, surpassing the importance of fine-tuning parameters or selecting algorithms. Correct thalamus and red nucleus segmentation play vital roles in ensuring accurate pathway computation.

**Conclusion:**

This study highlights the potential for automation in fiber tracking algorithms for tcFUS therapy, but acknowledges the ongoing need for expert verification and integration of anatomical expertise in treatment planning.

## 1. Introduction

Transcranial MR-guided focused ultrasound therapy (tcMRgFUS) offers precise thermal ablation of brain tissue. It effectively treats movement disorders like Parkinson's disease and essential tremor (ET) in a minimally invasive manner (Elias et al., 2016; Bond et al., 2017). The specific target area for tremor treatment is the ventral intermediate nucleus (VIM) of the thalamus, which acts as a synaptic “hub” mainly for the contralateral (but to a certain extent also the ipsilateral) cerebellothalamic tracts (CTTs) and the pallidothalamic tracts (PTTs; Gallay et al., 2008). The VIM cannot be localized in standard clinical magnetic resonance imaging (MRI); thus, attempts have been made to develop better thalamic segmentation procedures: either based on functional connectivity, specific magnetic resonance (MR) sequences aiming at improved contrast within the thalamus or using diffusion tensor imaging (DTI)-based tractography to provide increased accuracy to localize the VIM (Tourdias et al., 2014; Fasano et al., 2016; Akram et al., 2018; Dallapiazza et al., 2019; Shepherd et al., 2020; Purrer et al., 2021). The therapeutic ablation target, located at the inferior/lateral border of the thalamus, is quite small and necessitates a precision of approximately 0.5 mm. It is crucial to avoid major fiber tracts such as the corticospinal tract (CST) and the medial lemniscus (ML), which are in close proximity to the VIM and the target bundles (the CTT and the PTT), and must not be included in the ablation. Although diffusion tractography is prone to poor image resolution, high noise in the signal and distortion artifacts in sequences (Maier-Hein et al., 2017), fiber tracking algorithms can be utilized to better understand target areas and risk zones in the brain that require careful delineation (Fasano et al., 2016). This information greatly assists in the planning process, both prior to and during the procedure, by establishing safety margins and accurately calibrating the ablation positions according to the patient's unique anatomy.

We propose that it is essential to reconstruct the target bundles and the fiber bundles that require protection with utmost accuracy, aligning them with individual morphological images instead of relying solely on atlas-based coordinates. This becomes even more significant due to the ongoing debate regarding whether the most efficient structure to be targeted lies within the VIM or the respective bundles.

The problem of qualitative or quantitative evaluation of fiber tracking algorithms is well known. The aim of this article is neither to show that one fiber tracking algorithm is better than the other nor to establish new methods or metrics for comparing different methods. Rather we aim to answer the question whether a full automatic pipeline is possible at all or whether a manual, fine-tuned fiber tracking and segmentation is needed, which is time-consuming and requires expert knowledge of complex open-source neuroimaging tools. To this end, we examine whether fiber tracking results are highly dependent on the algorithm, pipeline or degree of automation used, or whether they are quite robust and, in combination with an automated calculation of the standard treatment coordinate (Jameel et al., 2022), can provide a robust planning basis for essential tremor therapy (ETT).

## 2. Materials and methods

TcMRgFUS therapy at the University Hospital Bonn is performed within the neurosurgical operating area. The therapy system employed consists of the INSIGHTEC Exablate Neuro system with a helmet-like phased array ultrasound transducer containing 1024 elements operating at a frequency of 660kHz, in conjunction with a 3T MRI scanner (Discovery MR750w, GE Healthcare, Chicago, IL, USA). The patient's head is securely immobilized using a dedicated MR-compatible stereotactic frame affixed to the helmet transducer. T1w overview images are used for co-registration with the preoperative image data. The standard treatment coordinate is manually determined first according to the manufacture suggestions and neurosurgical practice on a manually anterior commissure (AC) posterior commissure (PC) reoriented T1-weighted image as described in the last part of Section 2.5.2. During the treatment, the target region is manually optimized using the coordinates of the tracked fiber bundles on axial, coronal and sagittal images by applying individual, subtherapeutic, low-intensity test US pulses, followed by neurological examination to assess the single sonication effects. The fiber bundles probable locations (relative to the AC/PC and the midline) are used to move the temperature center in the presumed optimal direction (target bundles) and avoid no-go areas (CST, ML). Once a target is identified to be free of side effects when raising the temperature core to 50°C and gaining (temporarily) tremor control, the region is thermally ablated with higher energy pulses to reach 57°C to 60°C.

In the following subsections we describe the used data sets, the definition of the used anatomical regions of interest (ROIs) as well as the manual and the automatic planning pipelines. In addition, we describe the metrics we used for the evaluation.

### 2.1. Data sets

The data set used in this study consists of planning, treatment, and follow-up data for 45 patients who underwent tcMRgFUS treatment for ET at University Hospital Bonn. The components of a full patient data set are summarized in Table 1.

**Table 1:** Components of a complete patient data set used in this study.

Planning data	T1 MPRAGE planning image (1x1x1 mm^3^, 3D sequence) acquired on a 3T Philips Medical Systems Achieva scanner
	DWI planning data (2x2x2 mm^3^, 56 gradient directions, b-value = 1200) acquired on the same scanner
	fiber tracts of the CTT, PTT, CST, and ML from manual FSL pipeline used for therapy planning at University Hospital Bonn (see Section 2.4)
Treatment data	log data from Insightec Exablate Neuro system (Insightec Ltd, Israel) including treatment coordinates, temperatures, and affine registration matrix of treatment coordinates to T1 planning image
Follow-up data	T1 MPRAGE at 6 months after treatment (1x1x1 mm3, 3D sequence) acquired on a 3T Philips Medical Systems Achieva scanner
	manual segmentation of the visible lesion
Data from AFT-	standard coordinate for treatment from automatic pipeline (see Section 2.5.2)
based pipeline	fiber tracts of the CTT, PTT, CST, and ML from automatic planning pipeline (see Section 2.5.6)

The full data set was available for 23 patients. For two other patients, one was missing the tracked fibers of the CTT and the other, the ML. For 15 patients, only planning image data (no fiber tracts) and treatment data were available. For the remaining five patients, only planning image data were available.

### 2.2. tcFUS treatment data

The treatment log data was used to estimate the accuracy of the automatically predicted standard coordinate (see Section 2.5.2). We did not consider all ablation points but only those where a maximum average temperature of at least 55°C was reached, which is considered therapeutic. This can be more than one position per patient. The treatment positions were registered to the T1 planning coordinate system via the registration matrix determined during treatment (automatic registration followed by manual correction of the treating physician).

### 2.3. Anatomical definition of seed, include, and exclude ROIs

Table 2 shows the ROIs required for fiber tracking. Although we chose the same anatomical landmarks for the manual and the automatic planning pipelines, only for the automatic pipeline, the precentral gyrus was used as the seed region for both the CTT and the CST. As fiber tracking algorithms are usually non-bijective (start at A and end at B, start at B and do not end at A), it is much easier to track the respective bundle in its entirety with a comparatively much larger seed region and determine the borders of the bundles more precisely. However, the probabilistic approach used in the manual pipeline leads to a large number of different fibers being determined per seed point anyway.

**Table 2:** Anatomical ROIs used for fiber tracking of the CTT, the PTT, the CST, and the ML.

**CTT (FSL)**	**CTT (AFT)**	**PTT (FSL)**	**PTT (AFT)**	**CST (FSL)**	**CST (AFT)**	**ML (FSL/AFT)**
Dentate nucleus (seed)	Contralateral dentate nucleus (include)	Globus pallidus interna (seed)	Globus pallidus interna (seed)	Cerebral peduncle (seed)	Ipsilateral cerebral peduncle (include)	Midbrain portion of the medial lemniscus (seed)
Ipsilateral superior cerebellar peduncle (include)	Contralateral superior cerebellar peduncle (include)	Thalamus	Frontal part of thalamus	ipsilateral posterior limb of internal capsule (include)	Ipsilateral posterior limb of internal capsule (include)	Ipsilateral postcentral gyrus (include)
Contralateral red nucleus (include)	Ipsilateral red nucleus (include)			Ipsilateral thalamus (exclude)	Ipsilateral thalamus (exclude)	ipsilateral thalamus (include)
Contralateral thalamus (include)	Ipsilateral thalamus (include)			Ipsilateral precentral gyrus (include)	precentral gyrus (seed)	
Contralateral precentral gyrus (include)	Precentral gyrus (seed)					

Note that the anatomical description “contralateral/ipsilateral” is used in relation to the respective seed ROI.

ROI, region of interest; CTT, cerebellothalamic tract; PTT, pallidothalamic tract; CST, corticospinal tract; ML, medial lemniscus; FSL, FMRIB software library; AFT, adapted fiber tracking.

### 2.4. Manual planning pipeline

Aligning the image data along the line connecting the AC and PC not only provides a standardized view but also provides the basis for calculating the standard target coordinate for tcMRgFUS treatment. Therefore, an AC-PC orientation was manually set by reorienting the T1-MPRAGE. These reorientation parameters were also applied to all derived probability maps that represent the fiber tracts (see Section 2.4.2).

#### 2.4.1. Preprocessing

DTI images were preprocessed using FMRIB software library (FSL) (Smith et al., 2004). First, we corrected for the susceptibility distortions by applying the “topup” toolbox on the reverse phase encoding *b* = 0 image. This created a field corrected mask that we used to correct for eddy currents and motion using the “eddy” toolbox. Bayesian estimation and crossing fiber modeling along the principal diffusion directions were calculated using the bedpost toolbox in order to perform probabilistic tractography (Hernández et al., 2013). After the standard coordinates for treatment were determined on anatomical T1- MPARGE images, we further preprocessed the images. First, we skull-stripped and non-linearly transformed the individual T1-MPRAGE images of each patient to the Montreal Neurological (MNI) space atlas (Mori et al., 2008; Oishi et al., 2008). We obtained ROIs of different brain structures (see Section 2.3) in MNI 1-mm space in order to use them for fiber tractography. While the ROI of the thalamus was obtained from subcortical segmentation of the T1-MPRAGE of each individual, the other ROIs were non-linearly warped to the individual space to create the tracts in individual anatomical space. We also registered the T1-MPRAGE images to the diffusion space in order to apply the transformation matrix to the seed, include, and exclude ROIs for probabilistic tractography.

#### 2.4.2. Fiber tracking using FSL

Probabilistic tractography was performed using the default parameters of “probtrackx2,” which is part of the FSL diffusion toolbox FDT (Behrens et al., 2007) as exemplified in the study by Ferreira et al. (2021). All fiber tracts were normalized by dividing through total streamline counts reaching from each voxel of seed to target. Furthermore, we applied a threshold to the probability maps to remove false-positive streamlines, details are explained in Section 2.6. Normalized and thresholded tracts were inserted in the statistical parametric mapping pipeline with T1-MPRAGE to localize the tracts.

### 2.5. Automatic planning pipeline

#### 2.5.1. Summary of the automatic pipeline

**Figure 1:**
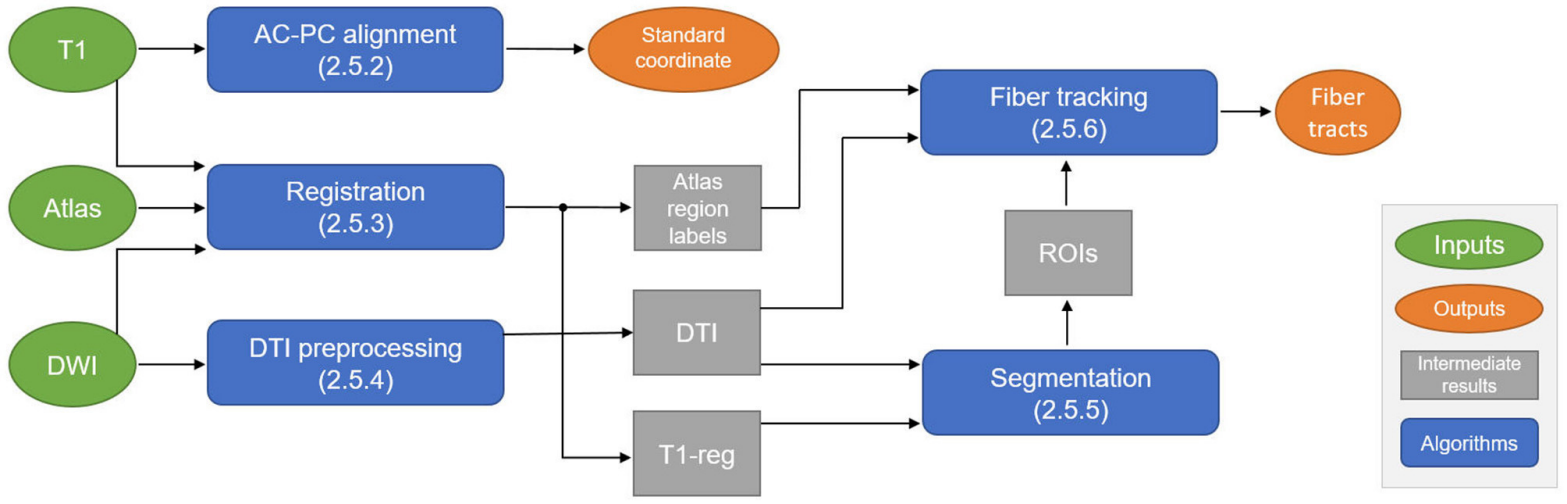
Schematic overview of the automatic planning pipeline to compute the standard coordinate for treatment and fiber tracts. DWI, diffusion-weighted imaging; AC, anterior commissure; PC, posterior commissure; DTI, diffusion tensor imaging; ROI, region of interest.

The overall data flow and algorithmic components of the proposed automatic planning pipeline are shown in Figure 1. Details on each step are given in Sections 2.5.2, 2.5.6. In the first step, the T1 image is automatically reformatted to the standardized AC-PC view, from which the geometric standard coordinate for treatment can be derived. For fiber tracking, the T1 image is registered to the diffusion-weighted imaging (DWI) space, where all the following steps are performed. The registration step also includes a deformable atlas registration to the patient data to gain anatomical region labels of the whole brain, which is used for local parameter adaptation during fiber tracking. The DWI data are preprocessed to extract a DTI. The registered T1 image and the DTI image are the input for a DL-based ROI segmentation algorithm that generates seed, include, and exclude ROIs for fiber tracking. These ROIs, the deformed atlas brain regions, and the DTI data are used to perform the final fiber tracking of the target bundles (CTT, PTT) and the no-go areas (CST, ML).

#### 2.5.2. DL-based AC-PC alignment and standard target coordinate

The AC and the PC (see Section 2.4 for an explanation) were detected using a 3D U-Net (Çiçek et al., 2016). To this end, the landmarks were manually annotated in the ETT cohort using markers. To develop the algorithm, a subset of 30 cases was used for training, 5 cases were used for validation during training, and 10 cases were used for evaluation. For training of the segmentation model, the markers were converted to masks and dilated. The 3D U-Net was then trained to predict the AC and PC marker blobs based on the T1 MPRAGE image. From the predicted masks, markers for the AC and the PC were extracted as the center of gravity of the predicted blob for the AC and the PC. The model was trained using the Dice loss function (Milletari et al., 2016) with learning rate of 10^−4^ with a batch size of 5 until convergence of the training loss, while the best-performing parameter state was tracked based on the Jaccard score on the validation data.

Using the predicted positions of the AC and the PC, the T1 image was rotated in the axial and sagittal plane so that the AC and the PC were in the same axial plane and on a vertical axis parallel to the voxel grid. To account for large rotations in the coronal plane (around the axis defined by the AC and the PC), the transformed image was registered to an AC-PC-aligned atlas. The rotation within the coronal plane was then applied to the transformed image to generate the final standardized AC-PC view.

The standard target coordinate was calculated based on the AC-PC reoriented image: anterior to the PC by 25% of the AC-PC distance, 1 mm superior, 14 mm lateral left/right (depending on the treated side) as typically performed at University Hospital Bonn. Different research groups may use slightly deviating definitions of the standard coordinate and the definition has evolved over time (Jameel et al., 2022).

#### 2.5.3. Registration

We register all available data sets, including the MNI atlas, that we used fiber tracking and for generating training data to the diffusion-weighted images. T1 data sets are rigidly registered to the diffusion MRI data; thereby, as a distance measure, we used normalized gradient fields. The atlas (for details, see Section 2.5.6) is registered to the diffusion-weighted data in a deformable way: after an initial rigid registration, the atlas is registered in a nonparametric way (distance measure: normalized gradient fields, optimization algorithm L-bfgs) using a multilevel approach.

#### 2.5.4. DTI preprocessing

We propose to supersample the data to an isotropic target voxel size with a higher order filter. This supersampling does not add any information to the image but allows for a simple trilinear interpolation in the later tracking phase. As proposed by Hahn et al. (2006), we use a Lanczos-3 filter in the spatial domain, which is a good compromise between computational speed and filter accuracy. In addition to spatial resampling, we smoothed the data with a Gaussian filter. Finally, we calculated the diffusion tensors using the well-known Stejskal–Tanner equation.

#### 2.5.5. DL-based ROI segmentation

All ROIs that we used as seed regions or include/exclude regions for fiber tracking were segmented using DL, as proposed by Hänsch et al. (2022). The segmentation algorithm was developed on a subset of 30 cases for training and 5 cases for validation (the same split as detailed in Section 2.5.2). A total of 24 ROIs (see Section 2.3 for details) is required per patient to generate the target bundle and no-go areas so that the manual annotation of the whole training set would be very time-consuming. Therefore, the majority of the training segmentations was created automatically via a deformable atlas registration (see Section 2.5.3), which propagates the ROIs that we already computed for the manual pipeline; see Section 2.4.1. For a subset of the ROIs (thalamus, ventricles, pre- and postcentral gyrus), the accuracy of the registered ROIs was found to be unsatisfactory. Therefore, these ROIs were segmented fully manually (thalamus) and using semiautomatic algorithms and tools for ventricles (Hahn et al., 2001) and gyri (Weiler and Hahn, 2015) to generate training data. In total, three U-Nets were trained: (1) rough brain extraction based on the T1 image and the registered brain outline, (2) ventricles based on the T1 image, and (3) the remaining ROIs based on the T1 image and the color-coded direction map derived from DTI; see Hänsch et al. (2022) for details.

For (1) and (2), standard 3D U-Nets (Çiçek et al., 2016; with 3 and 4 resolution levels, respectively) were trained. For (3), an anisotropic U-Net (Chlebus et al., 2022) was trained to allow for a larger receptive field in the axial plane. Moreover, the two input images (the T1 image and the color-coded direction map) were processed in separate convolutional pathways before combining the extracted feature maps at the lowest resolution level in the U-Net architecture. All models were trained using the Dice loss function. For the multi-structure segmentation in (3), with highly varying volumes of structures to be segmented, a weighting scheme was applied that weights each structure with its inverse volume throughout the training set. This way, smaller structures are assigned a higher weight and are less likely to be omitted by the model.

#### 2.5.6. Adapted fiber tracking

Fiber tracking was performed using a fiber tracking algorithm that locally adapts its parameters to specific regions of the JHU-MNI-ss white matter atlas (Klein et al., 2015). The atlas, also known as the “Eve atlas” (Mori et al., 2008; Oishi et al., 2008), is based on T1-MRI data from 152 healthy volunteers and consists of 176 regions. For each position during fiber tracking, the covering atlas region was calculated, for which optimized parameter sets were defined in advance and used locally to calculate the fiber segments within that region.

If the fibers are computed for all seed points, the tracking process is repeated if a minimum number of fibers has not been tracked or if the maximum number of iterations has not been reached. In our experiments, the minimum number of fibers per bundle was set to 100, and the maximal number of iterations has been set to 500, except for the CTT, where up to 2,500 iterations are allowed as it is challenging for the tractography algorithm to find fibers running through the very small red nucleus and the thalamus at the same time.

### 2.6. Evaluation

We registered all data sets including derived image data and coordinates to the T1 space of each patient. Due to the overall small cohort size, we evaluated each measurement on as many cases as possible for which the required data were available.

We compared the average distance in mm between actual treatment positions extracted from the log data (registered to T1) and the calculated standard coordinate (a) manually set in SPM (statistical parametric mapping) and (b) automatically computed by our DL approach. These data are available for 40 patients. For comparing the fiber bundles tracked by both approaches, we compare the distances between the fiber bundles (represented by the probability maps) and (a) the center of the lesion (which has been manually segmented by an experienced neurologist using 6 months' postoperative MRI data), (b) the average treatment point, and (c) the calculated standard coordinate. These data are available for 25 patients (one missing FSL-based CTT, one missing FSL-based ML). Note that for a fair comparison, the probability maps delivered by both approaches have to be thresholded in such a way so that the resulting bundles have nearly the same diameter and show only fibers that are defined as highly likely by each method. This can be achieved by scaling each probability map to [0,1] and using the following heuristically determined thresholds: For any probability map derived by the FSL approach, we take voxel values into account only if their value is at least 0.1. Considering the adapted fiber tracking (AFT) approach, probability maps representing the CTT, the PTT, or the CST are thresholded by 0.2, and maps representing the ML are thresholded by 0.5. If using the same threshold for both approaches or at least the same thresholds for same bundles, the fiber bundle tracked by the FSL approach would always be thinner with respect to the diameter. Even when the threshold is close to 0, the bundle is still thinner because FSL is not able to track fibers at the borders of the 3 fiber bundles very well. Additionally, we examine the influence of the clinician's decision whether the treatment points were selected close to the suggested automatic standard coordinate or whether the treatment points were farther away; see Section 3.1. For that purpose, we define the average deviation of *K* treatment points *p*(*i, j*) (corresponding to patient *j*) from the calculated standard coordinate stdCoord(*j*) for a single patient *j* by


(1) $$\begin{array}{l} \text{dev}\left(j\right)=\frac{1}{K}\overset{K}{\underset{i=1}{\sum }}|p\left(i,j\right)-\text{stdCoord}\left(j\right)|, \end{array}$$

where |*p*(*i, j*)−stdCoord(*j*)| denotes the Euclidean distance between *p*(*i, j*) and the standard coordinate.

Then, the average deviation for a given set of *N* patients where for *M* different *j*, dev(*j*) fulfills the condition *cond* is defined as


(2) $$\begin{array}{l} {\text{dev}}_{cond}=\frac{1}{M}\overset{N}{\underset{\begin{array}{c} j=1\text{ cond}\left(\text{dev}\left(j\right)\right) \end{array}}{\sum }}\text{dev}\left(j\right). \end{array}$$

As the VIM is located at the lateral border of the thalamus, it is interesting to examine how distances behave depending on the amount of lateral/medial change between calculated standard coordinate and final treatment points. The deviation of the calculated standard coordinate from the AC/PC line is always 14.0 mm as defined. Thus, for patient *j* the absolute deviation of *K* treatment points *p*(*i, j*) from the calculated standard coordinate with respect to their lateral/medial change is defined as


(3) $$\begin{array}{l} \text{xdev}\left(j\right)=\frac{1}{K}\overset{K}{\underset{i=1}{\sum }}|p_{x}\left(i,j\right)-14|, \end{array}$$

where *p*_*x*_(*i, j*) denotes the *x*-component of *p*_*i*_ corresponding to patient *j*.

Correspondingly, we define the average deviation for a given set of *N* patients where for *M* different *j*, dev_*x*_(*j*) fulfills the condition *cond* as


(4) $$\begin{array}{l} {\text{xdev}}_{cond}=\frac{1}{M}\overset{N}{\underset{\begin{array}{c} j=1\text{ cond}\left(\text{xdev}\left(j\right)\right) \end{array}}{\sum }}\text{xdev}\left(j\right). \end{array}$$

## 3. Results

**Figure 2:**
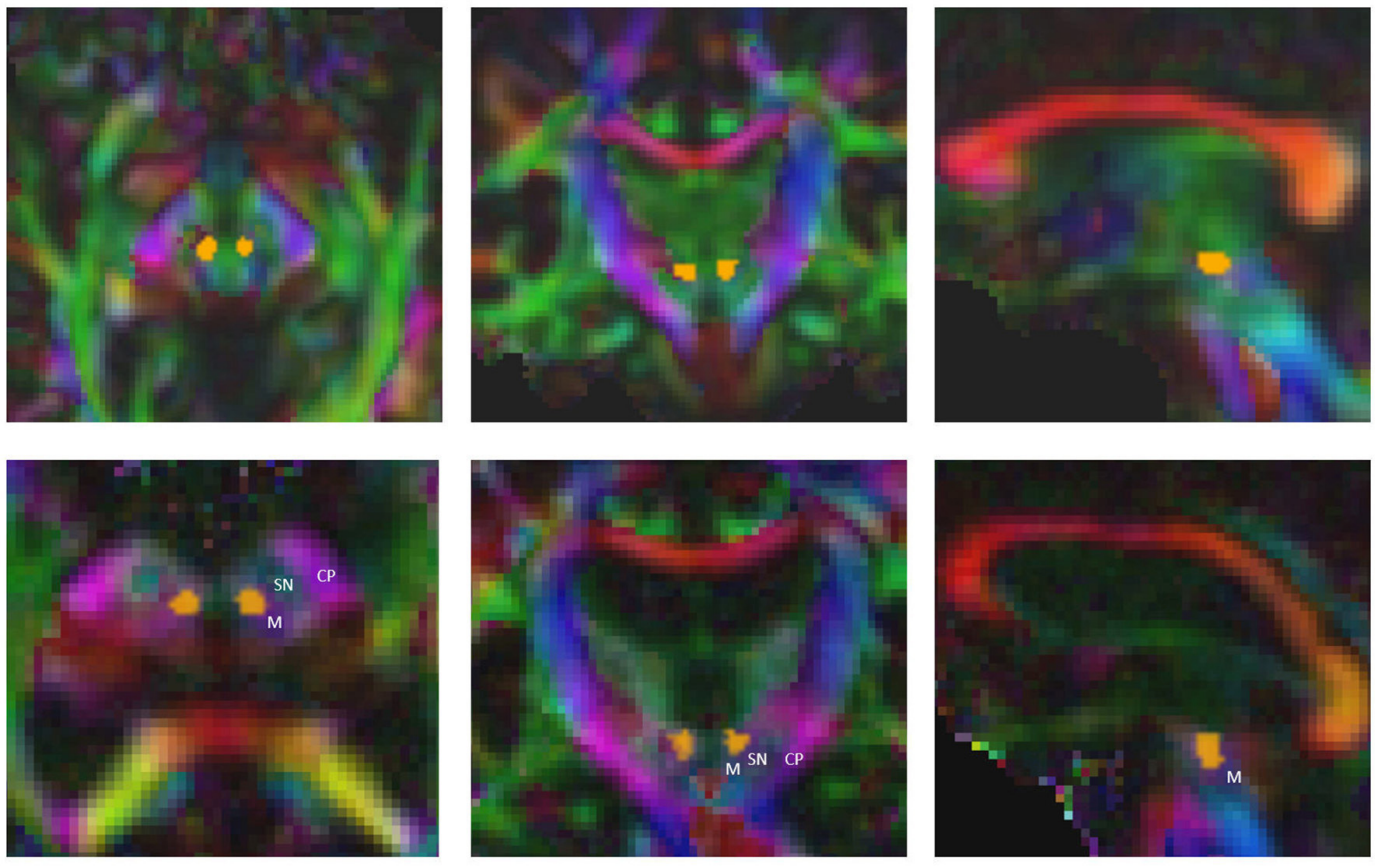
Precise segmentation of the red nucleus constitutes the basis for tracking the correct part of cerebellothalamic tract. M, midbrain; SN, substantia nigra; CP, cerebral peduncle.

The time required to determine both the standard coordinate and the fiber bundles using the FSL-based pipeline amounts to at least 4 h for a single patient (AMD(R) Ryzen threadripper 3960x 24-core processor). The computational time needed for all the necessary processing steps in the AFT pipeline is approximately 6 minutes for a single patient [using the following system: Intel(R) Core(TM) i7-4770K central processing unit (CPU) @ 3.50 GHz, NVIDIA TITAN Xp]. With the automatic AFT-based pipeline, the only requirement is to select the location of the DICOM data and the treatment side of the patient. The rest is automated, resulting in the employee's engagement time or waiting time being nearly identical to the required computational time. Out of the approximately 6 minutes, the registration, preprocessing, segmentation, and automatic AC-PC alignment take about 2.5 min (DL segmentation requires an additional 3 min if there is no graphics card in the PC and the computation is performed on the CPU). The remaining 3.5 min are needed for fiber tracking: the CST and the ML can usually be calculated within seconds, while the CTT requires more time. This time could be reduced if the required minimum number of 100 fibers per bundle were reduced to 50 and/or if the maximum number of iterations for CTT tracking were decreased from 2,500. The CTT is computationally intensive because we use the entire precentral gyrus as a seed region, resulting in tracking significantly more fibers per iteration compared to using a small ROI such as the red nucleus (see Figure 2).

### 3.1. Standard coordinate

The mean euclidean distance of the standard coordinate to the treatment positions (>55°C) across 40 patients is 1.90 ± 0.72 mm (automatic computation of standard coordinate) and 3.15 ± 1.45mm (manually defined standard coordinate). The treatment was therefore on average performed within the range of 2 mm to the automatically computed target location. A study on deep brain stimulation found ablation positions deviating by more than 2 mm (in the AC-PC plane) from the optimal location to be associated with poorer tremor control (Papavassiliou et al., 2004). Therefore, it is recommended also for tcMRgFUS to aim for an ablation position within a 2-mm range of the optimal target location (Focused Ultrasound Foundation, 2019). Our automatically computed standard coordinate fulfills this requirement, if we assume the treated locations to be optimal with respect to tremor control.

### 3.2. ROI segmentation

A prerequisite for the fiber tracking step is an accurate automatic segmentation of fiber tracking ROIs. Tables 3, 4 show the mean and the standard deviation of the Dice score for all individual ROIs on the 10 test cases for DL algorithm development. In the case of registered “reference” segmentations, this must be considered a quality estimate only, as the registration itself may be inaccurate. The highest mean Dice score is achieved for the thalamus, which is an important ROI for all considered tracts. For the cerebral peduncle, the resulting Dice scores are low (<0.5), which may be explained by the very small size of this ROI. Overall, all required ROIs are detected by the automatic segmentation pipeline.

**Table 3:** Mean and standard deviation of the Dice score for all computed regions of interest with manually curated reference segmentations on *N* = 10 test cases of the deep learning algorithm development.

	**Thalamus**	**Precentral gyrus**	**Postcentral gyrus**	**Lateral ventricle**	**3rd ventricle**	**4th ventricle**
Dice, left side	0.86 ± 0.04	0.74 ± 0.05	0.71 ± 0.09	0.82 ± 0.09		
Dice, right side	0.88 ± 0.03	0.64 ± 0.07	0.68 ± 0.04	0.82 ± 0.09		
Dice					0.69 ± 0.16	0.57 ± 0.18

**Table 4:** Mean and standard deviation of the Dice score for all computed regions of interest with registered, uncurated reference segmentation on *N* = 10 test cases of the deep learning algorithm development.

	**Cereb. peduncle**	**Dentate nucleus**	**Globus pall. interna**	**Post limb int. capsule**	**Red nucleus**	**Sup. cereb. peduncle**	**Midbrain portion of ML**
Dice, left side	0.34 ± 0.21	0.81 ± 0.04	0.75 ± 0.05	0.81 ± 0.04	0.66 ± 0.12	0.78 ± 0.04	0.80 ± 0.05
Dice, right side	0.44 ± 0.26	0.79 ± 0.06	0.74 ± 0.08	0.79 ± 0.05	0.61 ± 0.90	0.77 ± 0.06	0.80 ± 0.04

### 3.3. Fiber tracts

**Figure 3:**
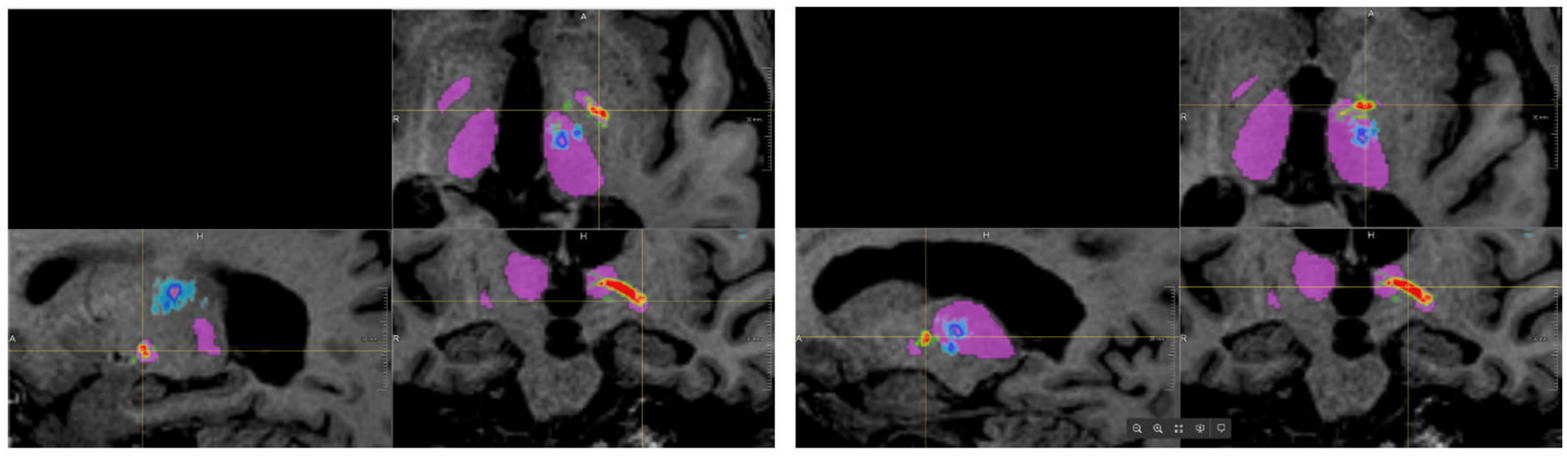
Results of tracking the pallidothalamic tract using both approaches (blue/cyan: FSL, red/yellow/green: adapted fiber tracking).

When using the FSL pipeline, the PTT was not restricted to the area between the globus pallidus interna and the thalamus but could extend throughout the brain, especially toward the cortex. Consequently, many false-positive fibers were computed, which would result in very small (or zero) distances between the standard coordinate and the PTT. In the AFT pipeline, however, we restricted the pathways of the PTT between the two ROIs, especially the anterior part of the thalamus. This allows for a reconstruction of the PTT that closely matches the descriptions and results demonstrated by Kwon et al. (2021; see Figure 3) and could be utilized for therapy planning. Thus, we decided not to compare the results of the PTT in more detail as it appears to be of little help due to the significant differences of both approaches.

**Figure 4:**
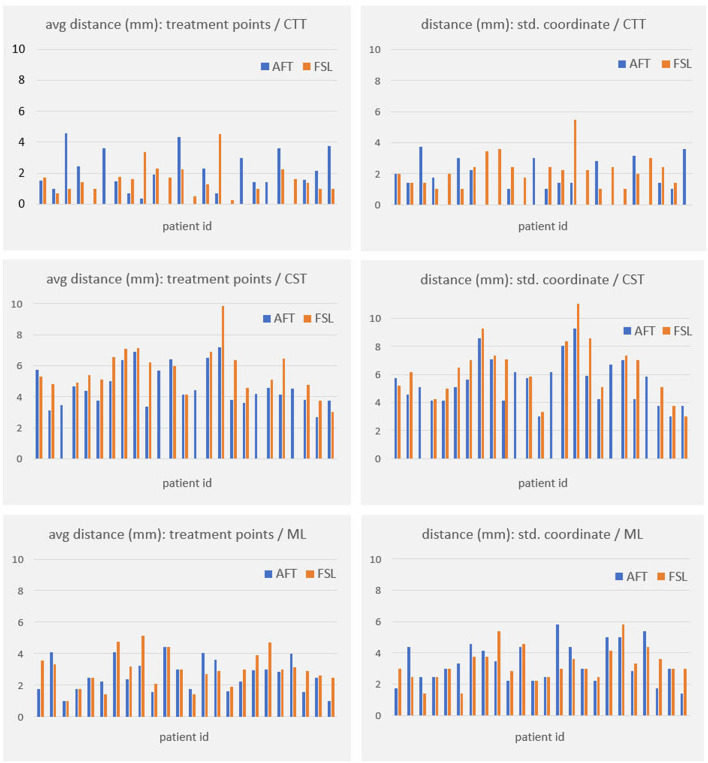
Distances (in mm) between core of fiber bundle and (a) average treatment point (plots in left column) and (b) calculated standard coordinate (plots in right column). As the CTT is the target bundle for treatment, distances are clearly smaller for this specific bundle compared to no-go bundles of the CST and the ML. CTT, cerebellothalamic tract; CST, corticospinal tract; ML, medial lemniscus; AFT, adapted fiber tracking; FSL, FMRIB software library.

**Figure 5:**
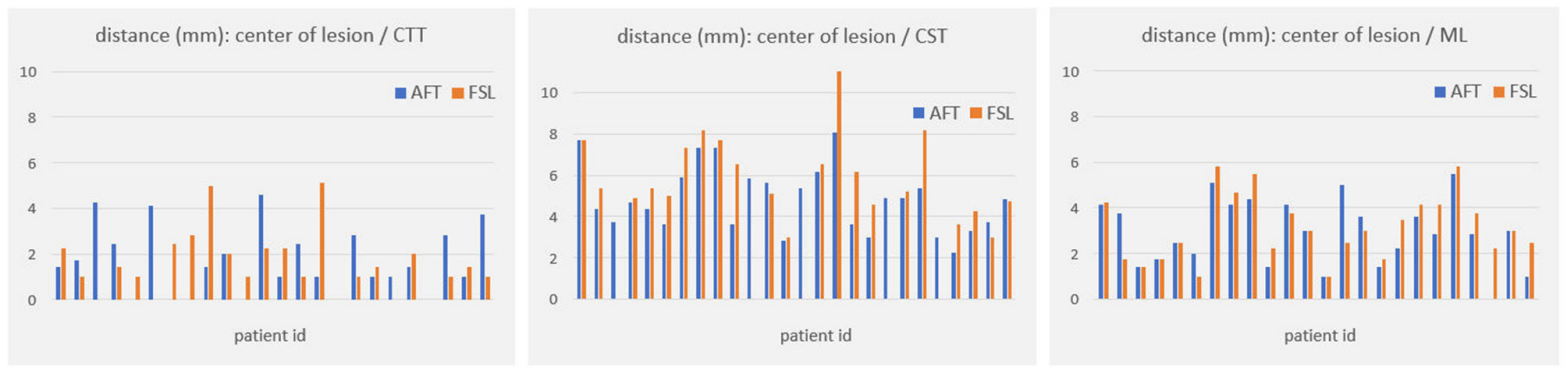
Distances (in mm) between core of fiber bundle and lesion center (6 months' postoperative). CTT, cerebellothalamic tract; CST, corticospinal tract; ML, medial lemniscus; AFT, adaptive fiber tracking; FSL, FMRIB software library.

For all other bundles, distances between the borders of calculated fiber bundles and the treatment points and the calculated standard coordinate and the center of the lesion for each individual patient are compared in Figures 4, 5. All plots show that both approaches yield very similar results, especially for the no-go bundles of the CST and the ML. It should be noted that the thresholding as described in Section 2.6 shrinks the fiber bundles in such a way that their extent is comparable for both fiber tracking approaches. Thus, even for the target bundle, the CTT, distances greater than zero can occur. For the CTT, some minor deviations between both approaches can be detected; see also Table 5: the average distance between the CTT and the center of the lesion is 1.56 mm (FSL) and 1.68 mm (AFT); between the CTT and the average treatment point, 1.40 mm (FSL) and 1.74 mm (AFT); and between the CTT and the calculated standard coordinate, 2.01 mm (FSL) and 1.42 mm (AFT).

**Table 5:** Average distances (in mm) between core of fiber bundle and (a) center of lesion (6 months' postoperative), (b) average treatment point, and (c) calculated standard coordinate.

	**AFT / FSL dev_≥0_**	**AFT / FSL dev_≥2_**	**AFT / FSL dev_ < 2_**	**AFT / FSL xdev_≥1_**	**AFT / FSL xdev_ < 1_**
Center of lesion ↔ CTT	1.68 / 1.56	1.80 / 1.58	1.55 / 1.53	**1.75 / 1.38**	1.53 / 1.69
Center of lesion ↔ CST	4.83 / 4.76	4.75 / 5.11	4.81 / 4.33	4.95 / 4.29	**4.38 / 5.48**
Center of lesion ↔ ML	2.90 / 3.11	2.70 / 3.20	3.07 / 3.04	2.82 / 2.85	2.99 / 3.42
Avg treatment point ↔ CTT	**1.74 / 1.40**	**2.04 / 1.47**	1.44 / 1.32	**1.88 / 1.45**	1.52 / 1.30
Avg treatment point ↔ CST	4.65 / 4.53	4.46 / 4.84	4.74 / 4.16	4.81 / 4.15	4.44 / 5.02
Avg treatment point ↔ ML	2.63 / 2.95	2.39 / 2.95	2.83 / 2.94	2.57 / 2.74	2.69 / 3.19
Standard coordinate ↔ CTT	**1.42 / 2.01**	1.67 / 2.03	**1.16 / 1.99**	**1.43 / 1.80**	**1.38 / 2.18**
Standard coordinate ↔ CST	5.48 / 5.08	5.63 / 5.71	5.23 / 4.38	**5.67 / 4.60**	5.25 / 5.68
Standard coordinate ↔ ML	3.36 / 3.25	3.36 / 3.33	3.35 / 3.18	3.41 / 3.23	3.29 / 3.27

Measurements are shown for both fiber tracking approaches (AFT and FSL) and are also split up into patient groups where distances between the calculated standard coordinates and treatment points are considered. Numbers are in bold if the two algorithms yield results that differ by more than 20%.

AFT, adapted fiber tracking; FSL, FMRIB software library; CTT, cerebellothalamic tracct; CST, corticospinal tract; ML, medial lemniscus.

As the average lateral/medial deviation of the treatment points from the standard coordinate is 1.00 mm and as the average deviation of the standard coordinate from the treatment points is 2 mm, we used those two values for splitting the groups for a more detailed consideration: dev_<2_ and xdev_<1_ represent average distances between treatment points and standard coordinates that are closer together than for dev_≥2_ and xdev_≥1_ (details are provided in Section 2.6). If averaging all 9 measurements (i.e., the results of all 3 bundles times the 3 distances to the center, the treatment points, and the standard coordinate), then the difference between FSL and AFT is 13.6% in the case of considering only dev_≥2_; it is 11.85% for dev_<2_; the difference between FSL and AFT is 16.04% for xdev_≥1_ and 14.57% for xdev_<1_. Although the differences are not as significant as expected, this means that if one only considers patients for whom the later treatment points are closer to the calculated standard coordinate, both algorithms achieve even more similar results. In contrast, if the doctor takes a greater influence on the treatment points and defines them farther away from the automatically calculated standard coordinate, the results differ a little bit more. Note that the doctor's modifications are based exclusively on the FSL results. Thus, especially for the CTT target bundle, the values for the FSL algorithm are always a little bit better (=smaller) than those from the AFT algorithm when determining the distance to the treatment points and to the lesion center. The situation is different for the distance to the calculated standard coordinate, where the AFT algorithm consistently shows smaller distances. In this case, if only the distance between the CTT and calculated standard coordinate is considered, the difference between the two methods is greater than 20% for almost all groups (except for dev_≥2_, where the difference is 17.73%).

## 4. Discussion

One notable difference between both approaches is that the AFT-based approach computes continuous fiber tracts belonging to a specific fiber bundle. In the used FSL pipeline, which utilizes the red nucleus as a seed ROI, it does not generate continuous fibers that pass through all ROIs but, rather, a probability map where appropriate fiber portions need to be identified through clever thresholding.

Both the analysis of individual patients (Figures 4, 5) and the analysis of average distances show high similarities between the results. The largest difference occurs in patients where the treatment positions deviated at least 1 mm laterally/medially from the calculated standard position (i.e., xdev_≥1_). Here, four out of nine measurement results show a difference of more than 20%. In comparison, in all other cases, only 1 or a maximum of 2 out of 9 measurement results show a corresponding difference. However, it should be noted that the FSL-based results were used for optimizing the treatment points, and the treatment points were optimized based on these fiber tracts. Therefore, it was expected that the greatest difference would exist for xdev_≥1_, as this is where the relatively largest modifications were made by the doctors. Even when considering the average distance from the calculated standard coordinate to the center of the target bundle, the CTT (see Table 5), it can be observed that the AFT-based pipeline determined a distance of only 1.42 mm, whereas the FSL-based pipeline determined a distance of 2.01 mm.

The analysis of cases with larger deviations often revealed differences in segmented or registered ROIs. For example, the segmentation of the precentral gyrus in the MNI Atlas extends deep into the brain, not just on the cortical surface. This means that in the FSL pipeline, a fiber only needs to touch a voxel in a lower region to be counted. The subsequent path of the fiber is not taken into account by the pipeline. For instance, fibers from the CST or the CTT can easily drift into the postcentral gyrus.

**Figure 6:**
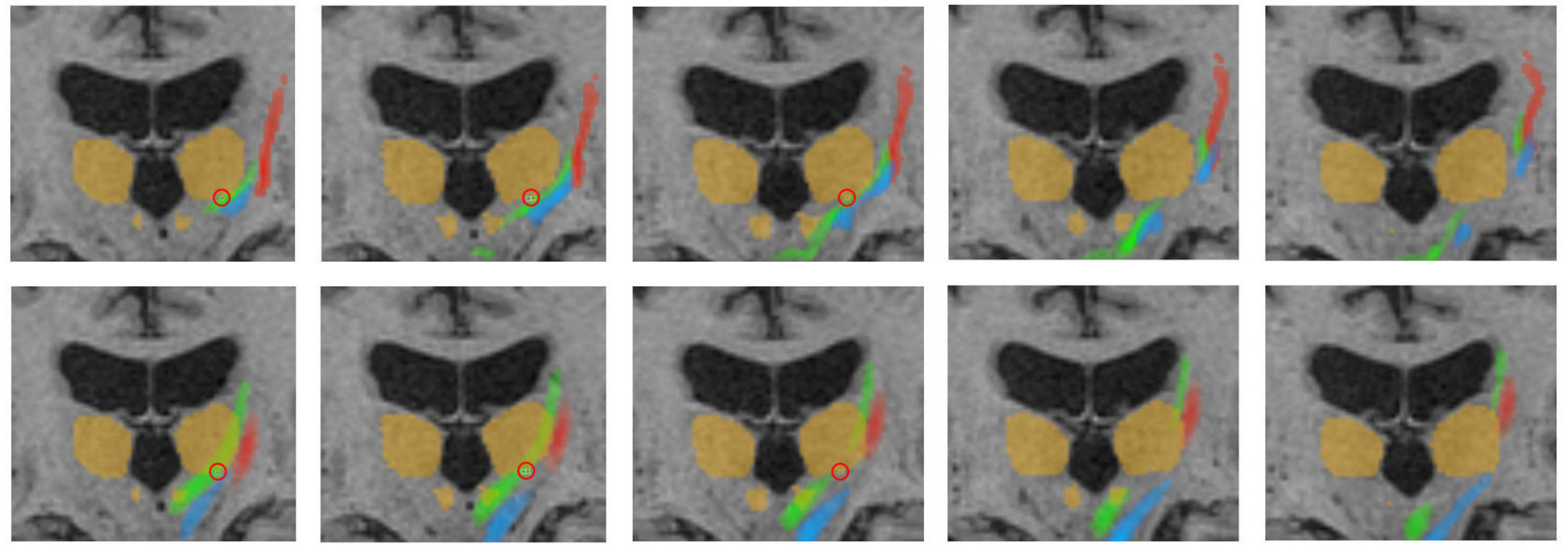
Representative fiber tracking results for the two pipelines. Green: CTT, blue: ML, red: CST. **Upper row**: FSL, **bottom row**: AFT. Treatment position and standard coordinate are marked by a red circle. The visualized thalamus and the red nuclei are identical in both rows for a better comparison of the fiber tracts; however, ROIs for fiber tracking have been differently determined for the manual and automatic planning pipeline as described in Sections 2.4.1 and 2.5.5. This explains, for example, the fact that only a part of the CTT (tracked by FSL) passes the red nucleus. CTT, cerebellothalamic tract; ML, medial lemniscus; CST, corticospinal tract; FSL, FMRIB software library; AFT, adapted fiber tracking; ROI, region of interest.

**Figure 7:**
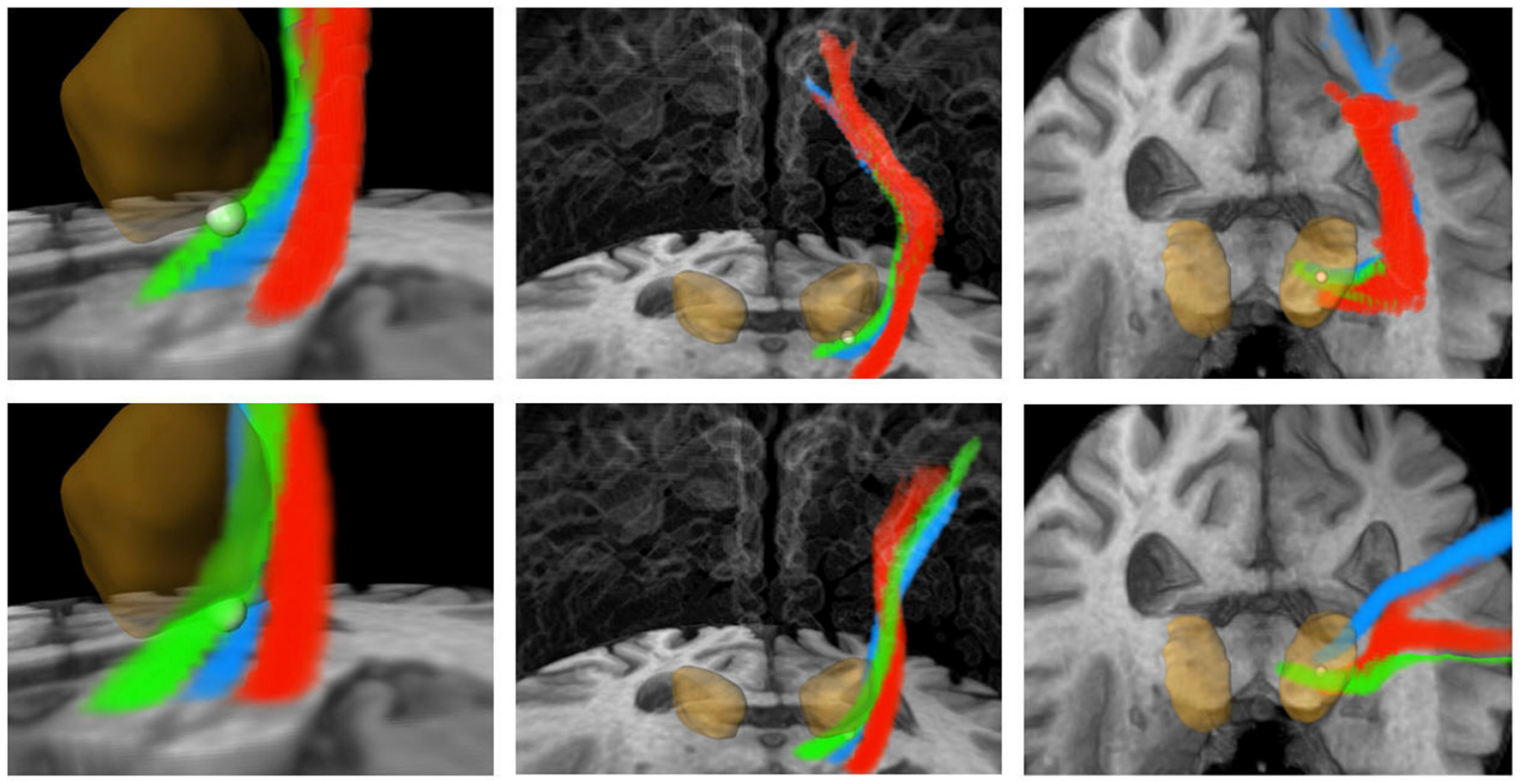
Three-dimensional visualization corresponding to Figure 6. Green: CTT, blue: ML, red: CST. **Upper row**: FSL, **bottom row**: AFT. The calculated standard coordinate is shown as a bright sphere. The DL-based segmented thalamus is identical in both rows for a better comparison of the fiber tracts. CTT, cerebellothalamic tract; ML, medial lemniscus; CST corticospinal tract; FSL, FMRIB software library; AFT, adapted fiber tracking; DL, deep learning.

Furthermore, precise segmentation is crucial not only for the very small red nucleus (see Figure 6) but also for the accurate segmentation of the thalamus. If the segmentation of the thalamus is slightly too large, the fiber tracking algorithm will quickly drift toward the internal capsule, where there is strong anisotropy, and mistakenly track parts of the pyramidal tracts. Conversely, if the segmentation of the thalamus is too small, the calculated standard coordinate may end up outside the thalamus, and only fibers that do not pass through the VIM will be computed (see Figures 6, 7).

By using a DL network, the segmentation can be significantly more robust and accurate. Another advantage of DL is reliability: while in our experiments, there were instances where the postcentral gyrus was incorrectly determined instead of the precentral gyrus during ROI-based segmentation (requiring subsequent manual correction), in all cases of DL-based segmentation, the correct gyrus was segmented. Note that the evaluation was conducted on a subset of data used for developing the DL algorithm as there was a very limited number of complete data sets available. However, it is important to mention that for most ROIs, there was no gold-standard reference segmentation available anyway. As an additional limitation it should be noted that during the treatment process, registration errors can occur in the treatment log data. This registration is typically performed to align the treatment position with the planning data using a registration matrix.

Our results have demonstrated that automatic fiber tracking and automatic determination of the standard coordinate are highly feasible. The AFT approach for automatically determining the fiber bundles required for ETT planning as well as the DL-supported calculation of the standard coordinate have shown that anatomically meaningful and plausible results can be achieved. Compared to a different, FSL-based pipeline where steps are manually performed or triggered and where the standard coordinate is manually determined, the results are generally quite similar in terms of measured distances to the target bundle, the standard coordinate, and the achieved lesion. This is particularly remarkable considering that the individual components differ algorithmically (preprocessing, fiber tracking algorithm). One reason for that is that the fiber bundles are strictly constrained by a number of narrowly defined ROIs. In addition, for both approaches, diffusion tensors were calculated, which allow for a robust reconstruction of fiber tracts.

However, in individual cases, the bundles may still deviate more from each other. Upon closer examination of these cases, it is often found that the ROIs were segmented differently. Here, the DL-based approach has a notable advantage over the registration-based approach, as it can typically produce more accurate segmentations.

Therefore, precise segmentation of the ROIs is extremely important, even more so than fine-tuning the fiber tracking parameters or choosing the algorithm, particularly if the algorithm relies on these ROIs. The treating physicians should never focus solely on the fiber tracts but always consider the ROIs used, ideally in combination with color-coded DTI images. A correct segmentation of the thalamus plays a crucial role in nearly all pathways, while the correct segmentation of the very small red nucleus is crucial for computing the CTT. Assessing the segmentation of the pre- and postcentral gyrus on axial slice images is not easy, especially if the images are not aligned to the AC/PC line. Checking those segmented gyri on a three-dimensional volume rendering (skull-stripped) is significantly easier and reduces errors.

A fully automated pipeline seems desirable both in the scientific and clinical context, as it saves the user a lot of time and manual work. The FSL-based pipeline takes approximately 4 hours to complete per patient, while the AFT-based approach takes an average of only 6 minutes per patient. In the clinical context, one would also want to enable manual control and correction of ROIs and fiber bundles to allow optimal treatment planning.

DL algorithms that learn the anatomical course of the fiber tracts themselves might provide additional safety and might help prevent continuity errors or drifting into areas with strong anisotropy. In particular, because the patients are neither tumor nor stroke patients, the fiber tracts are more similar, making it easier to create a valid training set. It is obvious that doctors should still verify these tracts using anatomical knowledge and landmarks.

## Data availability statement

The original contributions presented in the study are included in the article/supplementary material, further inquiries can be directed to the corresponding author. Requests to access these datasets should be directed to jan.klein@mevis.fraunhofer.de.

## Ethics statement

The studies involving humans were approved by Ethikkommission Bonn 314/18, Germany. The studies were conducted in accordance with the local legislation and institutional requirements. Written informed consent for participation was not required from the participants or the participants' legal guardians/next of kin in accordance with the national legislation and institutional requirements.

## Author contributions

JK: Conceptualization, Formal analysis, Methodology, Software, Validation, Visualization, Writing—original draft, Writing—review & editing. AG: Software, Writing—original draft, Writing—review & editing. NA: Software, Writing—review & editing. SR: Software, Writing—review & editing. NU: Writing—original draft, Writing—review & editing. VP: Data curation, Validation, Writing—review & editing. CS: Writing—review & editing. VB: Writing—review & editing. MK: Writing—review & editing. IR: Writing—review & editing. YS: Writing—review & editing. UW: Conceptualization, Supervision, Writing—review & editing. JJ: Conceptualization, Funding acquisition, Project administration, Supervision, Writing—review & editing.
